# Baseline triglyceride–cholesterol–body weight index and risk of incident cardiovascular disease: evidence from the CHARLS and ELSA cohorts

**DOI:** 10.3389/fnut.2026.1807288

**Published:** 2026-04-21

**Authors:** Peijian Wang, Ping Qiu, Beiyao Gao, Shan Jiang, Siyuan Wang

**Affiliations:** 1 Department of Rehabilitation Medicine, China-Japan Friendship Hospital, Beijing, China; 2 Department of Thyroid, Breast and Vascular Surgery, Zhenhai People's Hospital, Ningbo, Zhejiang, China

**Keywords:** cardiovascular disease, CHARLS, ELSA, nutritional status, triglyceride–cholesterol–body weight index

## Abstract

**Background:**

The triglyceride–cholesterol–body weight index (TCBI) has been proposed as a composite indicator of nutritional–metabolic status, yet evidence regarding its association with incident CVD in general aging populations and across different modeling strategies remains limited.

**Methods:**

We analyzed harmonized data from the China Health and Retirement Longitudinal Study (CHARLS: 2011–2018) and the English Longitudinal Study of Aging (ELSA: 2002–2018). Community-dwelling adults aged 45 years and older and free of CVD at baseline were included. Baseline TCBI was calculated as triglycerides × total cholesterol × body weight /1,000 and primarily analyzed as a log-transformed continuous variable. Incident CVD was defined as physician-diagnosed heart disease or stroke during follow-up. Cox proportional hazards models, restricted cubic spline analyses, and prespecified subgroup analyses were conducted separately in each cohort.

**Results:**

A total of 6,013 participants (CHARLS: 3,741; ELSA: 2,272) were included. In the fully adjusted model, higher baseline TCBI was significantly associated with incident CVD in both cohorts (CHARLS: HR = 1.216, 95% CI (1.092, 1.352); *P* < 0.001; ELSA: HR = 1.195, 95% CI (1.022, 1.396); *P* = 0.025) when modeled as a continuous variable. Quartile-based analyses were broadly consistent, although the pattern was not entirely monotonic, particularly in ELSA. Restricted cubic spline analyses showed a significant overall association without evidence of non-linearity in CHARLS, whereas neither the overall nor the non-linear association was statistically significant in ELSA. The results remained robust in lag analyses. However, the incremental predictive value of TCBI beyond conventional risk factors was minimal, with no significant improvement in discrimination or reclassification in either cohort.

**Conclusions:**

Higher baseline TCBI was associated with incident CVD in two large aging cohorts, especially when modeled as a continuous exposure. However, its incremental predictive value beyond conventional cardiovascular risk factors was minimal. TCBI may therefore serve as a complementary metabolic and nutritional indicator rather than a stand-alone predictive tool.

## Introduction

Cardiovascular disease (CVD) remains a leading cause of morbidity and mortality worldwide, particularly among aging populations (1). While traditional cardiometabolic risk factors are widely used for CVD risk assessment, they may not fully reflect the combined influences of nutritional status and metabolic reserve on long-term cardiovascular vulnerability (2, 3). Identifying simple indicators that integrate these dimensions is therefore of clinical and public health relevance.

The triglyceride–cholesterol–body weight index (TCBI), derived from circulating triglycerides (TG), total cholesterol (TC), and body weight (BW), has been proposed as a composite indicator of nutritional–metabolic status (4). Previous studies have shown that TCBI is associated with prognosis in patients with cardiovascular and cerebrovascular diseases, including heart failure and ischemic stroke (5–8), suggesting that it captures broader metabolic vulnerability beyond isolated lipid abnormalities.

However, translating these findings from clinical populations to community-dwelling aging populations remains challenging. Most existing studies have focused on clinical populations, and population-based findings have been inconsistent (5, 9). In addition, it remains unclear whether TCBI is best modeled as a continuous exposure or categorized into discrete groups, particularly across populations with different metabolic profiles.

To address these gaps, we examined the association between baseline TCBI and incident CVD in two nationally representative aging cohorts from China (CHARLS) and England (ELSA). Using harmonized data and identical analytical strategies, we aimed to clarify the relationship between TCBI and CVD risk and to explore potential non-linear and subgroup-specific associations.

## Materials and methods

### Study design and population

This study was based on harmonized data from CHARLS and ELSA, two nationally representative, population-based cohort studies of aging. Both cohorts included community-dwelling middle-aged and older adults (age ≥ 45). Baseline data were collected in 2011 for CHARLS and in 2002 for ELSA, with biennial follow-up surveys conducted through 2018.

Participants with prevalent CVD or stroke at baseline, missing baseline TCBI, missing follow-up information, or missing key covariates were excluded. Each cohort was analyzed separately using identical analytical strategies.

### Definition of the TCBI

The TCBI was calculated at baseline according to the following formula:


$$\begin{array}{l} \text{TCBI }=\;\frac{\text{TG }\left(\text{mg}/\text{dL}\right)\;\times \text{ TC }\left(\text{mg}/\text{dL}\right)\;\times \text{ BW }\left(\text{kg}\right)}{1000} \end{array}$$

Fasting blood samples were collected by trained personnel following standardized protocols in both cohorts. Serum TG and TC were measured using enzymatic methods, and body weight was obtained during physical examinations using calibrated instruments. Baseline TCBI was analyzed as a continuous variable. Given its right-skewed distribution, TCBI values were log-transformed [log (TCBI)] prior to analysis.

### Definition of the CVD

Incident CVD was defined as a self-reported physician diagnosis of heart disease (including angina, myocardial infarction, congestive heart failure, or other heart conditions) or stroke during follow-up. At each survey wave, participants were asked to confirm or refute any previously reported diagnoses; disputed reports were retrospectively corrected. This approach to outcome ascertainment has been widely applied and validated in prior longitudinal studies (2, 10). Time-to-event was defined as the interval between baseline assessment and the follow-up wave in which CVD was first reported. Because cardiovascular events were identified at discrete survey waves, the exact date of onset could not be determined; therefore, events were assigned to the corresponding follow-up interval. This method is consistent with previous cohort studies (2, 11).

### Covariates

Baseline covariates were obtained from standardized questionnaires and interviews and included age, gender (male or female), educational level, marital status, current smoking status (no or yes), and drinking status (never or ever). In the ELSA cohort, race was additionally included. Physical activity was assessed based on self-reported frequency of vigorous, moderate, and light activity. Participants were classified into four mutually exclusive categories according to the highest intensity performed at least once per week: vigorous, moderate (without vigorous activity), mild (without moderate or vigorous activity), and sedentary (12). Hypertension was defined as systolic blood pressure ≥140 mmHg, diastolic blood pressure ≥90 mmHg, or self-reported use of antihypertensive medication. Diabetes was defined as self-reported physician-diagnosed diabetes, fasting plasma glucose ≥7.0 mmol/L, or glycated hemoglobin (HbA1c) ≥6.5%. The weight-adjusted waist index (WWI) was calculated as waist circumference (cm) divided by the square root of body weight. WWI has been shown to be a reliable indicator of central obesity and is strongly associated with cardiometabolic morbidity and mortality (13).

### Statistical analysis

Continuous variables are presented as means ± standard deviations for normally distributed data or medians with interquartile ranges for skewed distributions, whereas categorical variables were expressed as frequencies and percentages. Baseline characteristics were compared across quartiles of the TCBI using one-way analysis of variance (ANOVA) or the Kruskal–Wallis test for continuous variables and the chi-square test for categorical variables, as appropriate.

The association between baseline TCBI and incident cardiovascular disease (CVD) was examined using Cox proportional hazards regression models, with follow-up time defined as the interval from baseline assessment to the first report of CVD during follow-up. TCBI was primarily analyzed as a log-transformed continuous variable because of its right-skewed distribution. In additional analyses, TCBI was categorized into quartiles to explore potential dose–response patterns. Hazard ratios (HRs) and 95% confidence intervals (CIs) were estimated using three progressively adjusted models. Model 1 was unadjusted. Model 2 was adjusted for age, sex, educational level, and marital status. Model 3 was further adjusted for WWI, smoking status, drinking status, physical activity, hypertension, and diabetes. In the ELSA cohort, ethnicity was additionally included as a covariate. The proportional hazards assumption was evaluated using Schoenfeld residuals.

Linear trends across TCBI quartiles were assessed by assigning the median value of each quartile and modeling it as a continuous variable. Restricted cubic spline functions with four knots were applied to evaluate potential non-linear associations between TCBI and incident CVD. Overall and non-linear associations were assessed using likelihood ratio tests comparing models with and without spline terms. Given the absence of a clear threshold effect, TCBI was primarily interpreted as a continuous exposure.

Prespecified subgroup analyses were performed according to age (< 60 and ≥60 years), gender, smoking status, drinking status, median value WWI (CHARLS:11.10; ELSA:10.84), physical activity (sedentary, mild, moderate, vigorous), hypertension and diabetes. Effect modification was evaluated by including interaction terms between TCBI and subgroup variables in the fully adjusted model.

A sensitivity analysis was conducted to evaluate the robustness of the findings. Specifically, participants who developed CVD within the first 2 years of follow-up were excluded to reduce potential reverse causation.

To assess the incremental predictive value of TCBI beyond conventional cardiovascular risk factors, time-dependent receiver operating characteristic (ROC) analyses were conducted at 5 years of follow-up. A baseline prediction model was similar to model 3. The predictive performance of models incorporating TCBI, the atherogenic index of plasma (AIP), and the triglyceride–glucose (TyG) index was compared with that of the baseline model. Improvements in model discrimination and risk classification were evaluated using the change in area under the ROC curve (ΔAUC), the integrated discrimination improvement (IDI), and the net reclassification improvement (NRI).

All analyses were conducted separately for the CHARLS and ELSA cohorts using identical analytical strategies. A two-sided *P*–value < 0.05 was considered statistically significant. Statistical analyses were performed using R software (version 4.3).

## Results

### Baseline characteristics

**Figure 1:**
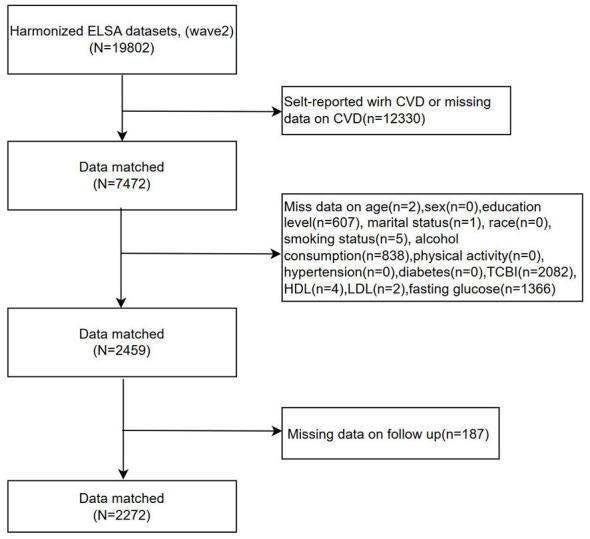
Flowchart of participant selection from the Harmonized ELSA.

**Figure 2:**
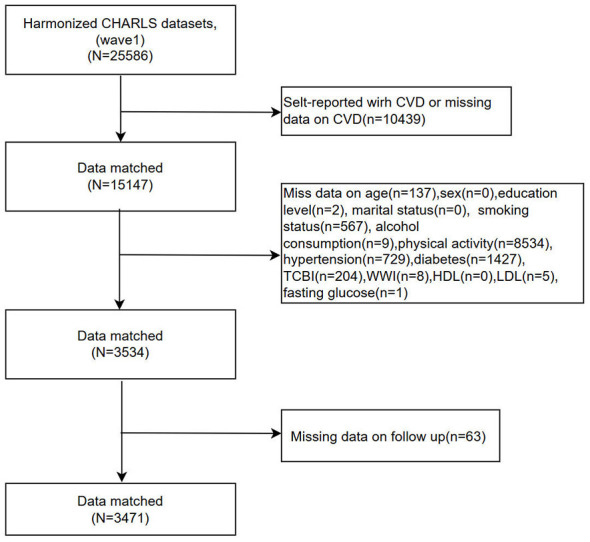
Flowchart of participant selection from the Harmonized CHARLS.

Figures 1, 2 show the participant selection processes for CHARLS and ELSA, respectively. A total of 6,013 participants (CHARLS: 3,741; ELSA: 2,272) were included. Baseline characteristics of both cohorts are presented in Tables 1, 2. To assess potential selection bias due to complete-case analysis, baseline characteristics were compared between included and excluded participants in each cohort (Supplementary Tables S1, S2). Several differences were observed, particularly in lifestyle factors and cardiometabolic conditions, although the overall distributions were broadly similar.

**Table 1:** The characteristics of participants according to quartiles of TCBI in CHARLS.

Variables	TCBI quartiles					*p*-value*^2^*
	Q1 *N* = 868*^1^*	Q2 *N* = 868*^1^*	Q3 *N* = 868*^1^*	Q4 *N* = 867*^1^*	Total*^1^*	
**Age (year)**	58.49 ± 10.29	58.42 ± 9.83	58.33 ± 9.37	57.26 ± 8.53	58.12 ± 9.54	0.022
Gender, *n* (%)						0.001
Male	425 (49%)	410 (47%)	365 (42%)	356 (41%)	1,556 (45%)	
Female	443 (51%)	458 (53%)	503 (58%)	511 (59%)	1,915 (55%)	
Education level, *n* (%)						>0.9
Less than lower secondary	795 (92%)	785 (90%)	791 (91%)	785 (91%)	3,156 (91%)	
Upper secondary & vocational training	65 (7.5%)	75 (8.6%)	71 (8.2%)	76 (8.8%)	287 (8.3%)	
Tertiary	8 (0.9%)	8 (0.9%)	6 (0.7%)	6 (0.7%)	28 (0.8%)	
Marital status, *n* (%)						0.084
Married or partnered	749 (86%)	771 (89%)	767 (88%)	782 (90%)	3,069 (88%)	
Other	119 (14%)	97 (11%)	101 (12%)	85 (9.8%)	402 (12%)	
**WWI**	10.91 (10.39, 11.49)	10.99 (10.48, 11.60)	11.19 (10.64, 11.76)	11.29 (10.83, 11.89)	11.11 (10.56, 11.71)	< 0.001
Current smoking, *n* (%)						0.010
Yes	286 (33%)	264 (30%)	240 (28%)	227 (26%)	1,017 (29%)	
No	582 (67%)	604 (70%)	628 (72%)	640 (74%)	2,454 (71%)	
Drinking, *n* (%)						0.2
Never	511 (59%)	526 (61%)	552 (64%)	530 (61%)	2,119 (61%)	
Ever	357 (41%)	342 (39%)	316 (36%)	337 (39%)	1,352 (39%)	
Physical activity						< 0.001
Sedentary	97 (11%)	69 (7.9%)	88 (10%)	96 (11%)	350 (10%)	
Mild	131 (15%)	169 (19%)	216 (25%)	214 (25%)	730 (21%)	
Moderate	229 (26%)	276 (32%)	259 (30%)	274 (32%)	1,038 (30%)	
Vigorous	411 (47%)	354 (41%)	305 (35%)	283 (33%)	1,353 (39%)	
Hypertension, *n* (%)						< 0.001
No	624 (72%)	572 (66%)	533 (61%)	425 (49%)	2,154 (62%)	
Yes	244 (28%)	296 (34%)	335 (39%)	442 (51%)	1,317 (38%)	
Diabetes, *n* (%)						< 0.001
No	796 (92%)	757 (87%)	735 (85%)	626 (72%)	2,914 (84%)	
Yes	72 (8.3%)	111 (13%)	133 (15%)	241 (28%)	557 (16%)	
CVD status during follow up, *n* (%)						< 0.001
No	720 (83%)	711 (82%)	679 (78%)	645 (74%)	2,755 (79%)	
Yes	148 (17%)	157 (18%)	189 (22%)	222 (26%)	716 (21%)	

^1^Mean ± SD; *n* (%); Median (Q1, Q3).

^2^One-way analysis of means; Pearson's Chi-squared test; Kruskal-Wallis rank sum test.

TCBI, triglyceride–cholesterol–body weight index; WWI, weight-adjusted waist index; CVD, cardiovascular disease.

**Table 2:** The characteristics of participants according to quartiles of TCBI in ELSA.

Variables	Triglyceride–Cholesterol–Body weight index(TCBI) quartiles					*p*-value*^2^*
	Q1 N = 568*^1^*	Q2 N = 568*^1^*	Q3 N = 568*^1^*	Q4 N = 568*^1^*	Total*^1^*	
**Age (year)**	63.18 ± 7.42	63.66 ± 7.25	63.39 ± 7.00	62.46 ± 6.79	63.17 ± 7.13	0.032
Sex, *n* (%)						< 0.001
Male	223 (39%)	256 (45%)	252 (44%)	324 (57%)	1,055 (46%)	
Female	345 (61%)	312 (55%)	316 (56%)	244 (43%)	1,217 (54%)	
Education level, *n* (%)						0.7
Less than lower secondary	174 (31%)	185 (33%)	203 (36%)	186 (33%)	748 (33%)	
Upper secondary & vocational training	304 (54%)	289 (51%)	278 (49%)	290 (51%)	1,161 (51%)	
Tertiary	90 (16%)	94 (17%)	87 (15%)	92 (16%)	363 (16%)	
Marital						0.086
Married or partnered	427 (75%)	428 (75%)	454 (80%)	452 (80%)	1,761 (78%)	
Other	141 (25%)	140 (25%)	114 (20%)	116 (20%)	511 (22%)	
**WWI**	10.52 (9.97, 10.97)	10.78 (10.31, 11.22)	10.95 (10.45, 11.35)	11.07 (10.70, 11.48)	10.84 (10.36, 11.28)	< 0.001
Race, *n* (%)						0.6
White	559 (98%)	561 (99%)	561 (99%)	564 (99%)	2,245 (99%)	
Non-white	9 (1.6%)	7 (1.2%)	7 (1.2%)	4 (0.7%)	27 (1.2%)	
Current smoking, *n* (%)						0.12
Yes	63 (11%)	70 (12%)	86 (15%)	85 (15%)	304 (13%)	
No	505(89%)	498(88%)	482(85%)	483(85%)	1,968(87%)	
Drinking, *n* (%)						0.2
Never	41(7%)	36(6%)	31(5%)	49(9%)	157(7%)	
Ever	527 (93%)	532 (94%)	537 (95%)	519 (91%)	2,115 (93%)	
Physical activity, *n* (%)						< 0.001
Sedentary	14 (2.5%)	16 (2.8%)	18 (3.2%)	24 (4.2%)	72 (3.2%)	
Mild	40 (7.0%)	49 (8.6%)	53 (9.3%)	81 (14%)	223 (9.8%)	
Moderate	287 (51%)	274 (48%)	302 (53%)	284 (50%)	1,147 (50%)	
Vigorous	227 (40%)	229 (40%)	195 (34%)	179 (32%)	830 (37%)	
Hypertension, *n* (%)						< 0.001
No	338 (60%)	284 (50%)	263 (46%)	219 (39%)	1,104 (49%)	
Yes	230 (40%)	284 (50%)	305 (54%)	349 (61%)	1,168 (51%)	
Diabetes, *n* (%)						0.002
No	558 (98%)	550 (97%)	546 (96%)	534 (94%)	2,188 (96%)	
Yes	10 (1.8%)	18 (3.2%)	22 (3.9%)	34 (6.0%)	84 (3.7%)	
CVD status during follow up, *n* (%)						0.004
No	465 (82%)	429 (76%)	440 (77%)	415 (73%)	1,749 (77%)	
Yes	103 (18%)	139 (24%)	128 (23%)	153 (27%)	523 (23%)	

^1^Mean ± SD; *n* (%); Median (Q1, Q3).

^2^One-way analysis of means; Pearson's Chi-squared test; Kruskal-Wallis rank sum test.

TCBI, triglyceride–cholesterol–body weight index; WWI, weight-adjusted waist index; CVD, cardiovascular disease.

Across TCBI quartiles, several consistent patterns were observed in both cohorts. With increasing TCBI, participants' age tended to decrease slightly, whereas WWI increased markedly (both *P* < 0.001). The prevalence of hypertension and diabetes rose progressively across TCBI quartiles in both cohorts. In CHARLS, hypertension increased from 28 in Q1 to 51% in Q4, and diabetes from 8.3 to 28%; in ELSA, hypertension increased from 40 to 61%, and diabetes from 1.8 to 6.0% (all *P* < 0.001). The proportion of participants who developed CVD during follow-up also increased across TCBI quartiles, from 17 in Q1 to 26% in Q4 in CHARLS and from 18 to 27% in ELSA. Physical activity distributions differed significantly across TCBI quartiles in both cohorts (both *P* < 0.001).

Notable cohort-specific differences were also observed. In CHARLS, the proportion of females increased with higher TCBI (48 in Q1 vs. 57% in Q4), whereas in ELSA the proportion of males increased (39 vs. 57%) (both *P* < 0.001). Current smoking decreased across quartiles in CHARLS (35 vs. 26%) but did not differ significantly in ELSA. Physical activity patterns also varied: in CHARLS, vigorous activity decreased from 47 to 33%, while mild activity increased from 15 to 25%; in ELSA, vigorous activity declined from 40 to 32%, whereas mild activity increased from 7.0 to 14%. Alcohol consumption decreased slightly across quartiles in CHARLS but did not differ significantly in ELSA. In ELSA, about 99% of participants were White, with no difference across quartiles.

### Association between baseline TCBI and incident cardiovascular disease

When TCBI was analyzed as a log-transformed continuous variable, higher baseline TCBI was significantly associated with incident CVD in both cohorts (Table 3). In the fully adjusted model, the association remained significant in CHARLS [HR = 1.216, 95% CI (1.092, 1.352); *P* < 0.001] and ELSA [HR = 1.195, 95% CI (1.022, 1.396); *P* = 0.025], indicating a consistent positive association between TCBI and incident CVD. Schoenfeld residual tests showed no violation of the proportional hazards assumption in either cohort (both *P* > 0.05).

**Table 3:** Association between triglyceride–cholesterol–body weight index (TCBI) and incident cardiovascular disease in the CHARLS and ELSA cohorts.

TCBI	CHARLS	*p*-value	ELSA	*p*-value
	HR (95%CI)		HR (95%CI)	
Model 1	1.297 (1.175, 1.432)	< 0.001	1.285 (1.113, 1.482)	< 0.001
Model 2	1.329 (1.202, 1.469)	< 0.001	1.296 (1.118, 1.502)	< 0.001
Model 3	1.216 (1.092, 1.352)	< 0.001	1.195 (1.022, 1.396)	0.025

HRs, Hazard ratios. CIs, confidence intervals.

Model 1: unadjusted. Model 2: adjusted for age, sex, education level, marital status. Model 3: further adjusted for weight-adjusted waist index, smoking status, drinking status, physical activity, hypertension, and diabetes. In the ELSA cohort, race was additionally adjusted for in Models 2 and 3.

Quartile-based analyses yielded similar results (Tables 4, 5). In CHARLS, compared with Q1, participants in Q4 had a significantly higher risk of incident CVD in the fully adjusted model [HR = 1.306, 95% CI (1.049, 1.626); *P* = 0.017], with a significant trend across quartiles (*P* for trend = 0.005). In ELSA, significantly higher risks were observed in Q2 [HR 1.340, 95% CI (1.035, 1.735); *P* = 0.026] and Q4 [HR 1.459, 95% CI (1.118, 1.903); *P* = 0.005], and the trend across quartiles was also significant (*P* for trend = 0.023). Overall, these findings were broadly consistent with the continuous analyses, although the quartile pattern was not entirely monotonic.

**Table 4:** Hazard ratios for incident cardiovascular disease according to quartiles of triglyceride–cholesterol–body weight index (TCBI) in the CHARLS cohort.

	Model 1		Model 2		Model 3	
	HR (95%CI)	*P*	HR (95%CI)	*P*	HR (95%CI)	*P*
Q1	Ref		ref		ref	
Q2	1.051 (0.840, 1.315)	0.665	1.047 (0.836, 1.310)	0.069	0.996 (0.795, 1.248)	0.971
Q3	1.298 (1.047, 1.609)	0.018	1.299 (1.047, 1.611)	0.018	1.188 (0.954, 1.478)	0.124
Q4	1.517 (1.232, 1.868)	< 0.001	1.560 (1.266, 1.923)	< 0.001	1.306 (1.049, 1.626)	0.017
*P* for trend	–	–	–	–	–	0.005

HRs, Hazard ratios. CIs, confidence intervals.

Model 1 was unadjusted. Model 2: adjusted for age, sex, education level, marital status. Model 3: further adjusted for weight-adjusted waist index, smoking status, drinking status, physical activity, hypertension, and diabetes.

*P* for trend was calculated by modeling the median TCBI value of each quartile as a continuous variable.

**Table 5:** Hazard ratios for incident cardiovascular disease according to quartiles of triglyceride–cholesterol–body weight index (TCBI) in the ELSA cohort.

	Model 1		Model 2		Model 3	
	HR (95%CI)	*P*	HR (95%CI)	*P*	HR (95%CI)	*P*
Q1	Ref		ref		ref	
Q2	1.488 (1.153, 1.920)	0.002	1.403 (1.087, 1.812)	0.009	1.340 (1.035, 1.735)	0.026
Q3	1.330 (1.026, 1.724)	0.031	1.277 (0.979, 1.647)	0.400	1.172 (0.899, 1.528)	0.257
Q4	1.637 (1.272, 2.107)	< 0.001	1.637 (1.272, 2.106)	0.014	1.459 (1.118, 1.903)	0.005
*P* for trend	–	–	–	–	–	0.023

HRs, Hazard ratios. CIs, confidence intervals.

Model 1 was unadjusted. Model 2: adjusted for age, gender, education level, marital status, race. Model 3: further adjusted for weight-adjusted waist index, smoking status, drinking status, physical activity, hypertension, and diabetes.

*P* for trend was calculated by modeling the median TCBI value of each quartile as a continuous variable.

### Dose–response relationship assessed by restricted cubic spline analysis

**Figure 3:**
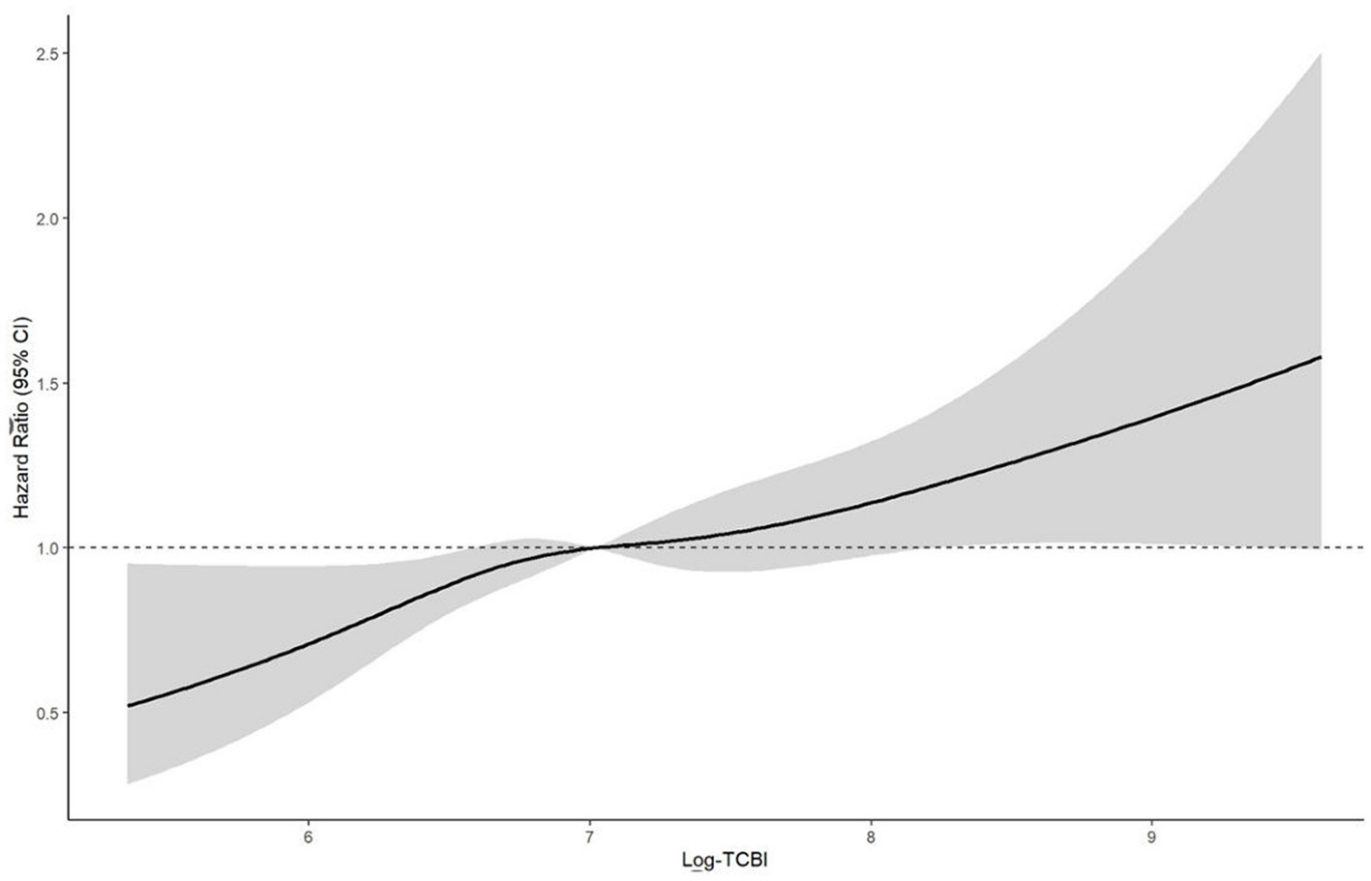
Restricted cubic spline analysis of the association between baseline triglyceride–cholesterol–body weight index (TCBI) and incident cardiovascular disease (CVD) in the CHARLS cohort. The solid line represents the estimated hazard ratio (HR = 1.0) was set at the median level of log-transformed TCBI. The model was adjusted for age, gender, educational level, marital status, weight adjusted waist index (WWI); smoking status, drinking status, hypertension, and diabetes.

**Figure 4:**
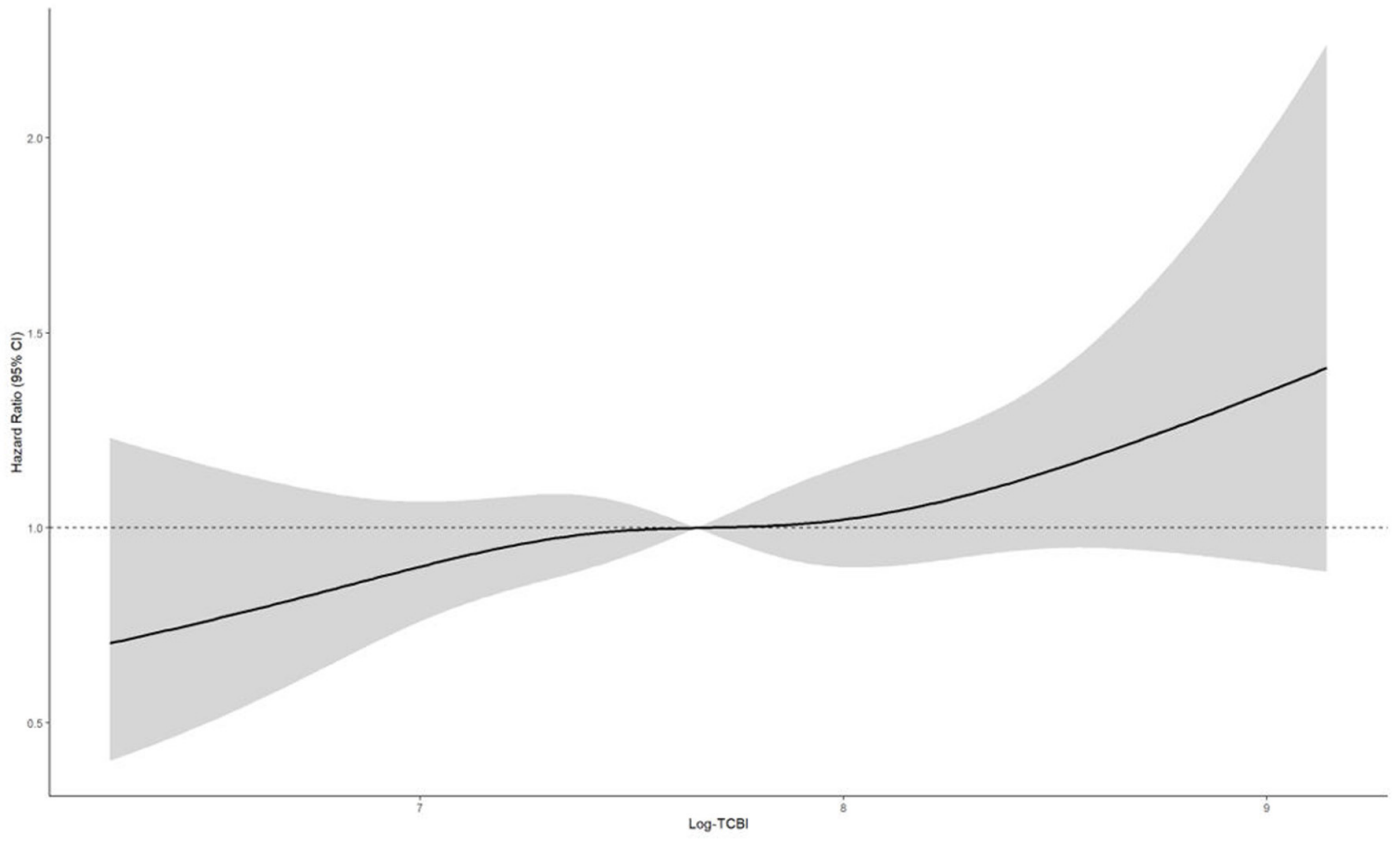
Restricted cubic spline analysis of the association between baseline triglyceride–cholesterol–body weight index (TCBI) and incident cardiovascular disease (CVD) in the ELSA cohort. The solid line represents the estimated hazard ratio (HR = 1.0) and the shaded area indicates the corresponding 95% confidence interval. The reference value (HR = 1.0) was set at the median level of log-transformed TCBI. The model was adjusted for age, sex, race, educational level, marital status, weight adjusted waist index (WWI); smoking status, drinking status, hypertension, and diabetes.

Restricted cubic spline analyses further supported these findings (Figures 3, 4). In CHARLS, the overall association was significant (*P* for overall = 0.004), but there was no evidence of non-linearity (*P* for non-linearity = 0.527). In ELSA, the overall association was of borderline significance (*P* for overall = 0.058), and no significant non-linearity was observed (*P* for non-linearity = 0.785). Thus, the spline analyses did not support a meaningful non-linear relationship in either cohort.

### Subgroup and interaction analyses

Subgroup analyses are shown in Tables 6, 7. In CHARLS, the association between higher TCBI and incident CVD remained significant in participants aged < 60 years, in both sexes, in non-current smokers, in never and ever drinkers, in those with sedentary or vigorous physical activity, in both WWI strata, and in those without hypertension or diabetes. In ELSA, significant associations were observed in participants aged < 60 years, men, non-current smokers, those with lower WWI, and those without hypertension or diabetes. However, no significant interaction was found for age, sex, smoking, drinking, physical activity, hypertension, or diabetes in either cohort. A significant interaction was observed only for WWI in ELSA (*P* for interaction = 0.039), with a stronger association in participants with lower WWI.

**Table 6:** Subgroup and interaction analyses of the association between baseline triglyceride–cholesterol–body weight index (TCBI) and incident cardiovascular disease in the CHARLS cohort.

Subgroup	Category	*n*	Events	HR (95% CI)	*P*-value	*P* for interaction
Age	< 60	2,079	356	1.26 (1.09, 1.46)	0.002	0.299
	≥60	1,392	360	1.15 (0.98, 1.36)	0.079	
Sex	Male	1,556	294	1.23 (1.04, 1.46)	0.014	0.892
	Female	1,915	422	1.21 (1.05, 1.40)	0.009	
Smoking	Non-current	2,454	526	1.25 (1.10, 1.42)	< 0.001	0.252
	Current	1,017	190	1.12 (0.91, 1.38)	0.288	
Drinking	Never	2,119	443	1.24 (1.08, 1.43)	0.003	0.491
	Ever	1,352	273	1.21 (1.01, 1.44)	0.034	
Physical activity	sedentary	350	86	1.46 (1.10, 1.95)	0.009	0.085
	Mild	730	179	1.26 (1.00, 1.58)	0.053	
	Moderate	1,038	216	1.04 (0.85, 1.28)	0.700	
	Vigorous	1,353	235	1.28 (1.07, 1.54)	0.008	
WWI	< median	1,735	313	1.24 (1.06, 1.46)	0.008	0.871
	≥median	1,736	403	1.19 (1.03, 1.38)	0.018	
Hypertension	0	2,154	352	1.29 (1.10, 1.51)	0.001	0.445
	1	1,317	364	1.14 (0.98, 1.33)	0.082	
Diabetes	0	2,914	581	1.25 (1.10, 1.42)	< 0.001	0.363
	1	557	135	1.14 (0.92, 1.40)	0.241	

CVD, cardiovascular disease; HR, hazard ratio; CI, confidence interval; WWI, weight-adjusted waist index.

HRs and 95% CIs were estimated using Cox proportional hazards models adjusted for age, sex, education level, marital status, weight-adjusted waist index, smoking status, drinking status, physical activity, hypertension, and diabetes, as appropriate.

Subgroup-specific hazard ratios represent the association per unit increase in log-transformed baseline TCBI.

WWI was dichotomized according to the cohort-specific median value.

*P* for interaction was derived from likelihood ratio tests comparing models with and without interaction terms between TCBI and the corresponding subgroup variable.

**Table 7:** Subgroup and interaction analyses of the association between baseline triglyceride–cholesterol–body weight index (TCBI) and incident cardiovascular disease in the ELSA cohort.

Subgroup	Category	*n*	Events	HR (95% CI)	*P*-value	*P* for interaction
Age	< 60	849	131	1.37 (1.01, 1.85)	0.044	0.110
	≥60	1,423	392	1.06 (0.89, 1.28)	0.505	
Sex	Male	1,055	274	1.27 (1.02, 1.57)	0.031	0.600
	Female	1,217	249	1.12 (0.89, 1.42)	0.323	
Smoking	Non-current	1,968	450	1.25 (1.06, 1.48)	0.009	0.518
	Current	304	73	0.91 (0.59, 1.41)	0.682	
Drinking	Never	157	45	1.59 (0.97, 2.62)	0.069	0.528
	Ever	2,115	478	1.15 (0.98, 1.36)	0.088	
Physical activity	Sedentary	72	23	0.97 (0.49, 1.95)	0.942	0.746
	Mild	223	47	1.22 (0.72, 2.07)	0.456	
	Moderate	1,147	263	1.20 (0.97, 1.50)	0.095	
	Vigorous	830	190	1.30 (0.99, 1.71)	0.060	
WWI	< median	1,136	226	1.44 (1.14, 1.82)	0.002	0.039
	≥median	1,136	297	1.02 (0.83, 1.25)	0.875	
Hypertension	No	1,104	191	1.48 (1.14, 1.92)	0.003	0.232
	Yes	1,168	332	1.05 (0.86, 1.28)	0.625	
Diabetes	No	2,188	495	1.20 (1.03, 1.41)	0.023	0.349
	Yes	84	28	0.90 (0.40, 2.06)	0.810	

CVD, cardiovascular disease; HR, hazard ratio; CI, confidence interval; WWI, weight-adjusted waist index. HRs and 95% CIs were estimated using Cox proportional hazards models adjusted for age, sex, education level, marital status, race, weight-adjusted waist index, smoking status, drinking status, physical activity, hypertension, and diabetes, as appropriate.

Subgroup-specific hazard ratios represent the association per unit increase in log-transformed baseline TCBI.

WWI was dichotomized according to the cohort-specific median value.

*P* for interaction was derived from likelihood ratio tests comparing models with and without interaction terms between TCBI and the corresponding subgroup variable.

### Lag analysis

In lag analyses excluding participants who developed CVD within the first 2 years of follow-up, the association remained significant in CHARLS [HR = 1.211, 95%CI (1.074, 1.368); *P* = 0.002] and ELSA [HR = 1.223, 95%CI (1.039, 1.439); *P* = 0.016], supporting the robustness of the main results.

### Incremental predictive value of metabolic indices

**Figure 5:**
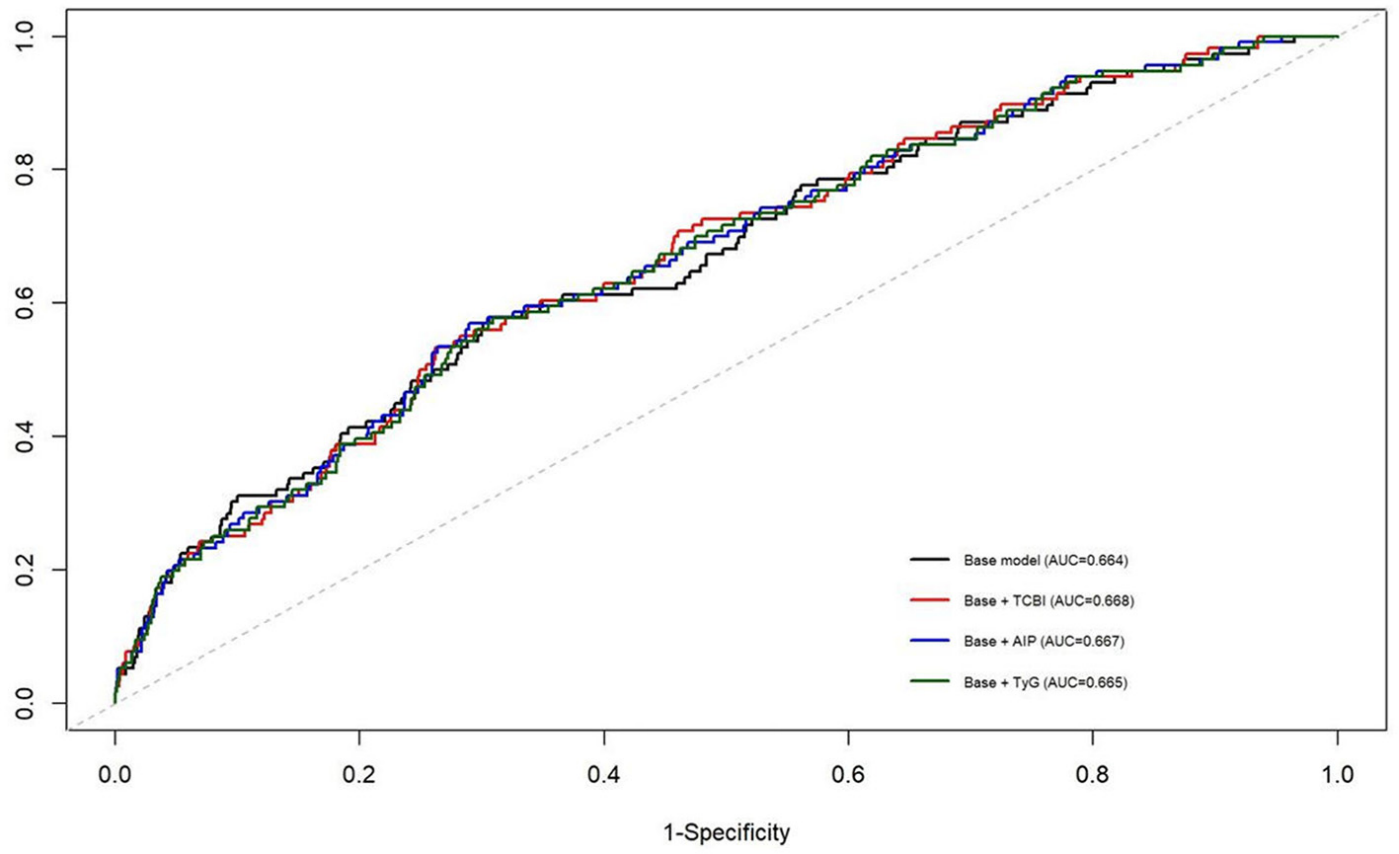
Time-dependent receiver operating characteristic (ROC) curve at 5 years for predicting incident cardiovascular disease in the ELSA cohort. The predictive performance of the fully adjusted baseline model was compared with models additionally including the triglyceride-cholesterol-body weight index (TCBI), the atherogenic index of plasma (AIP), or the triglyceride-glucose (TyG) index.

**Figure 6:**
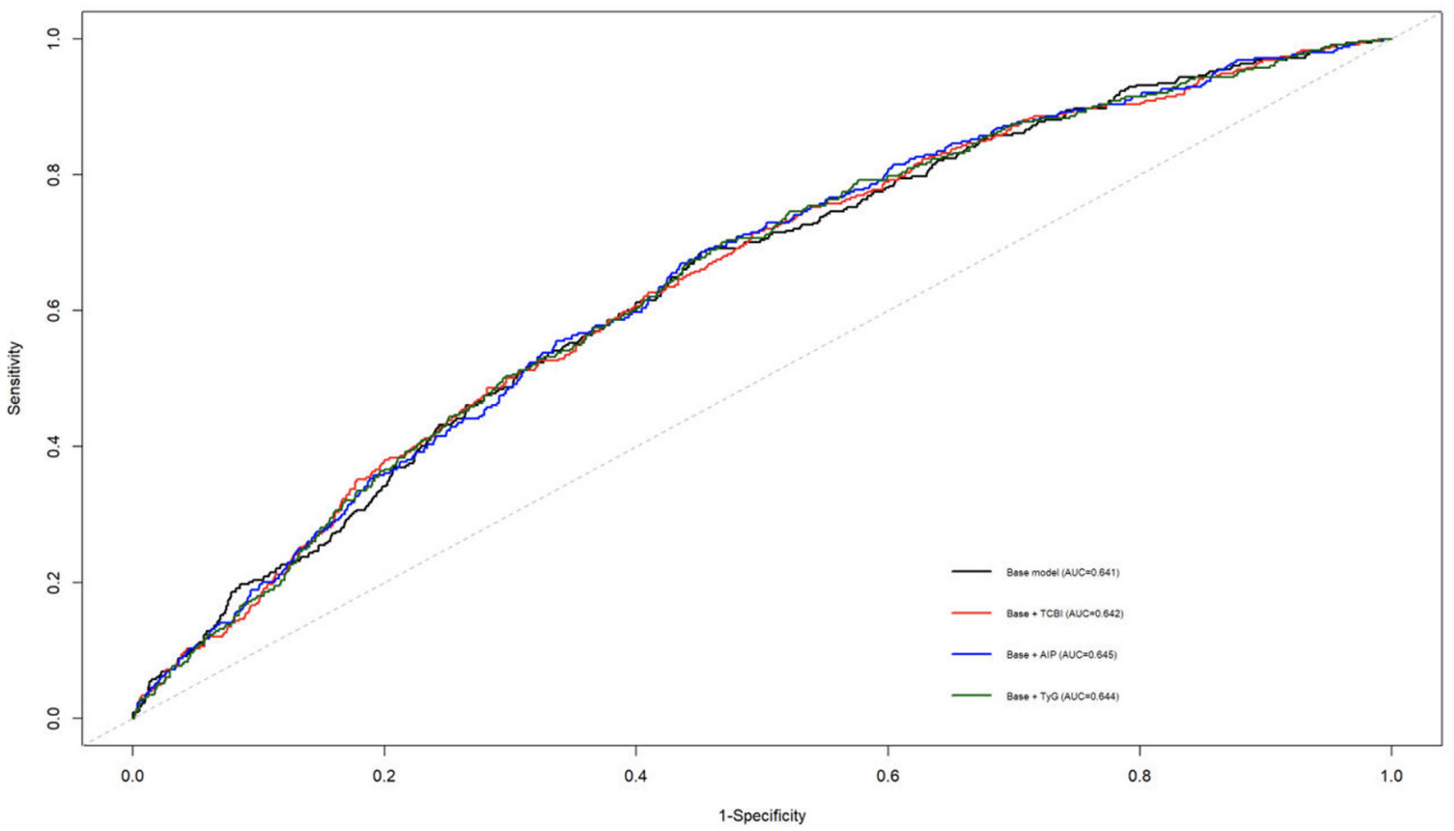
Time-dependent receiver operating characteristic (ROC) curve at 5 years for predicting incident cardiovascular disease in the CHARLS cohort. The predictive performance of the fully adjusted baseline model was compared with models additionally including the triglyceride-cholesterol-body weight index (TCBI), the atherogenic index of plasma (AIP), or the triglyceride-glucose (TyG) index.

To further evaluate the incremental predictive value of TCBI, time-dependent ROC analyses at 5 years were performed. In CHARLS, adding TCBI to the baseline model resulted in only minimal improvement in discrimination, with non-significant IDI and NRI. Similar findings were observed for AIP and TyG (see Table 8). Comparable results were also observed in ELSA, in which ethnicity was additionally included in the baseline model. ROC curves are shown in Figures 5, 6. Overall, although TCBI was associated with incident CVD, its incremental predictive value beyond conventional risk factors was modest.

**Table 8:** Incremental predictive value of metabolic indices for incident cardiovascular disease at 5 years in the CHARLS and ELSA cohorts.

Cohort	Marker	ΔAUC	*P*-value	IDI (95% CI)	*P*-value	NRI (95% CI)
CHARLS	TCBI	0.001	0.836	0.038 (−0.015, 0.087)	0.159	0.065 (−0.047, 0.190)
	AIP	0.003	0.398	0.061 (−0.001, 0.125)	0.070	0.151 (0.028, 0.272)
	TyG	0.002	0.560	0.070 (−0.016, 0.115)	0.890	0.137 (0.014, 0.236)
ELSA	TCBI	0.004	0.448	0.037 (−0.063, 0.129)	0.378	0.067 (−0.098, 0.260)
	AIP	0.002	0.555	−0.006 (−0.102, 0.106)	0.866	−0.029 (−0.192, 0.197)
	TyG	0.001	0.830	0.000 (−0.078, 0.118)	0.547	0.006 (−0.138, 0.199)

ΔAUC indicates the change in the area under the ROC curve after adding each metabolic index to the fully adjusted baseline model. The baseline model included age, sex, educational level, marital status, smoking status, alcohol consumption, WWI, physical activity, hypertension, and diabetes (race was additionally included in ELSA). AUC, area under the curve; IDI, integrated discrimination improvement; NRI, net reclassification improvement; TCBI, triglyceride–cholesterol–body weight index; AIP, atherogenic index of plasma; TyG, triglyceride–glucose index; WWI, weight-adjusted waist index; CVD, cardiovascular disease.

## Discussion

In this population-based study of two large aging cohorts from China and England, we found that higher baseline TCBI was associated with an increased risk of incident CVD when modeled as a continuous exposure. This association remained significant after adjustment for demographic characteristics, lifestyle factors, physical activity, and cardiometabolic comorbidities in both CHARLS and ELSA. Quartile-based analyses were broadly consistent with the continuous analyses, although the categorical pattern was less uniform across intermediate quartiles, particularly in ELSA. In addition, lag analyses excluding events occurring within the first 2 years of follow-up yielded similar results, supporting the robustness of the main findings. Overall, these findings support TCBI as a composite metabolic indicator associated with future CVD risk among community-dwelling adults aged 45 years and older.

The biological plausibility of this association is supported by the fact that TCBI integrates several key metabolic components closely linked to the pathogenesis of atherosclerosis and cardiometabolic dysfunction (14, 15). TG play a central role in lipid metabolism and are strongly associated with the formation of atherogenic lipoprotein particles. Elevated TG concentrations promote the generation of small dense LDL, which are more prone to oxidative modification and contribute to endothelial dysfunction and vascular inflammation (16). TC, another component of TCBI, represents the cumulative lipid exposure and facilitates lipid deposition within the arterial wall and promote macrophage activation and foam cell formation, which are key events in the initiation and progression of atherosclerosis (17). Body weight, the third component of TCBI, represents an important indicator of energy balance and metabolic reserve (18), and excess adiposity is closely linked to insulin resistance, dysregulated lipid metabolism, and chronic low-grade inflammation (18–20). Adipose tissue also functions as an endocrine organ that secretes adipokines and inflammatory mediators, including tumor necrosis factor-α and interleukin-6, which can impair endothelial function and promote vascular injury (21). By integrating TG, TC and BW into a single composite index, TCBI may capture the combined effects of dyslipidemia, metabolic imbalance, and chronic inflammation that contribute to cardiovascular disease development.

From a broader physiological perspective, TCBI may also reflect the interaction between metabolic status and nutritional physiology. Nutritional status has increasingly been recognized as an important determinant of cardiovascular health, particularly among aging populations (22). Adequate metabolic reserves contribute to metabolic stability and immune regulation, whereas metabolic imbalance and nutritional dysregulation may increase susceptibility to chronic systemic stress. Composite indices that incorporate metabolic and anthropometric parameters may therefore capture complex physiological interactions that cannot be adequately represented by single biomarkers. In this context, TCBI may serve as a simple integrative marker reflecting the combined influence of metabolic and nutritional factors on cardiovascular health (23).

The present findings also highlight the relationship between TCBI and other composite metabolic indices used for cardiometabolic risk assessment. Indices such as AIP and the TyG index have been widely investigated as markers of dyslipidemia and insulin resistance and have been associated with increased CVD risk in population-based studies (2, 24, 25). In the present study, however, adding TCBI to conventional risk factors resulted in only minimal improvement in discrimination and no significant improvement in reclassification, and similar findings were observed for AIP and TyG. These results suggest that, although TCBI is epidemiologically associated with incident CVD, its incremental predictive value beyond established cardiovascular risk factors is modest. Time-dependent ROC analysis and reclassification metrics (IDI and NRI) further confirmed this observation. Incorporating TCBI into the baseline risk model resulted in only minimal improvement in discrimination, and similar findings were observed for AIP and TyG. These results are not unexpected in population-based cohorts, where traditional risk factors such as age, smoking, hypertension, and diabetes already explain a substantial proportion of cardiovascular risk. Consequently, biomarkers that demonstrate significant epidemiological associations with disease outcomes may still yield only modest improvements in predictive discrimination (26). These findings suggest that the primary value of TCBI may lie in reflecting integrated metabolic and nutritional status rather than substantially improving established cardiovascular risk prediction models.

An additional observation of the present study was that continuous and categorical analyses were generally directionally consistent across the two cohorts, but quartile-based patterns were not entirely uniform. In CHARLS, higher TCBI quartiles were associated with progressively higher CVD risk, with a significant trend across quartiles. In ELSA, the quartile analyses also supported a positive association, and significant excess risks were observed in Q2 and Q4, although the intermediate pattern was not strictly monotonic. These findings suggest that categorization of a continuous metabolic marker may yield less stable estimates than continuous modeling, particularly when the exposure–outcome relationship is modest and sample distributions differ across populations (27–29). Consistent with this interpretation, restricted cubic spline analyses showed a significant overall association without evidence of non-linearity in CHARLS, whereas in ELSA neither the overall nor the non-linear spline term reached statistical significance. Taken together, these results do not support a meaningful threshold or non-linear pattern and favor interpreting TCBI primarily as a continuous exposure rather than relying on arbitrary categorical cut-points (30, 31).

Subgroup analyses suggested that the association between TCBI and incident CVD was more apparent in several lower-risk strata, including younger participants and those without hypertension or diabetes. This pattern may indicate that TCBI is more informative in individuals with a lower baseline cardiovascular burden, in whom metabolic imbalance may be detected before the influence of established vascular injury and competing risk factors becomes dominant. By contrast, in older adults and in those with major cardiometabolic comorbidities, the relative contribution of TCBI may be attenuated by more complex risk profiles and ongoing treatment. However, these findings should be interpreted cautiously, as formal interaction tests did not support significant effect modification for most subgroup variables in either cohort. The only significant interaction was observed for WWI in ELSA, with a stronger association among participants with lower WWI, suggesting that TCBI may capture additional metabolic risk even in individuals without more pronounced central adiposity (32). Overall, these results support a broadly stable association between TCBI and incident CVD across most demographic and clinical subgroups.

### Strengths and limitations

This study has several strengths. It was based on two large nationally representative aging cohorts from different geographic settings, enhancing the robustness and generalizability of the findings. We also applied consistent analytical strategies across cohorts, including continuous and categorical modeling, restricted cubic spline analyses, subgroup analyses, lag analyses, and assessment of incremental predictive performance. However, several limitations should be considered. Cardiovascular outcomes were based on self-reported physician diagnoses, which may introduce misclassification. TCBI was measured only at baseline, and residual confounding cannot be excluded. In addition, further validation in clinical cohorts or institutional datasets is warranted. Finally, although TCBI was associated with cardiovascular risk, its incremental predictive value beyond conventional risk factors was modest.

## Conclusions

In conclusion, higher baseline TCBI was associated with an increased risk of incident CVD in two large aging cohorts. This association was more consistently observed when TCBI was modeled as a continuous variable and remained robust across multiple analyses. However, the incremental predictive value of TCBI beyond conventional cardiovascular risk factors was modest. These findings suggest that TCBI may serve as a simple complementary indicator of metabolic and nutritional status in population-based cardiovascular research, rather than a stand-alone predictive tool.

## Data Availability

The datasets presented in this study can be found in online repositories. The names of the repository/repositories and accession number(s) can be found at: https://charls.pku.edu.cn and https://www.elsa-project.ac.uk.

## References

[1] Zhou M Wang H Zeng X Yin P Zhu J Chen W. et al. Mortality, morbidity, and risk factors in China and its provinces, 1990-2017: a systematic analysis for the global burden of disease study 2017. Lancet. (2019) 394:1145–58. doi: 10.1016/S0140-6736(19)30427-1 31248666 PMC6891889

[2] Li X Lu L Chen Y Liu B Liu B Tian H . Association of atherogenic index of plasma trajectory with the incidence of cardiovascular disease over a 12-year follow-up: findings from the ELSA cohort study. Cardiovasc Diabetol. (2025) 24:124. doi: 10.1186/s12933-025-02677-w 40108582 PMC11924681

[3] Celik E Cora AR Karadem KB. The Effect of Untraditional Lipid Parameters in the Development of Coronary Artery Disease: Atherogenic Index of Plasma, Atherogenic Coefficient and Lipoprotein Combined Index. J Saudi Heart Assoc. (2021) 33:244–50. doi: 10.37616/2212-5043.1266 34631402 PMC8480409

[4] Doi S Iwata H Wada H Funamizu T Shitara J Endo H. et al. A novel and simply calculated nutritional index serves as a useful prognostic indicator in patients with coronary artery disease. Int J Cardiol. (2018) 262:92–8. doi: 10.1016/j.ijcard.2018.02.039 29706396

[5] Maruyama S Ebisawa S Miura T Yui H Kashiwagi D Nagae A. et al. Impact of nutritional index on long-term outcomes of elderly patients with coronary artery disease: sub-analysis of the SHINANO 5 year registry. Heart Vessels. (2021) 36:7–13. doi: 10.1007/s00380-020-01659-0 32607637 PMC7788017

[6] Ishiwata S Yatsu S Kasai T Sato A Matsumoto H Shitara J . Prognostic effect of a novel simply calculated nutritional index in acute decompensated heart failure. Nutrients. (2020) 12:3311 doi: 10.3390/nu12113311 33137941 PMC7694067

[7] Xiao B Zhu J Han Y. Association of a novel nutritional marker, the triglyceride-cholesterol-body weight index, with 90-day unfavorable outcomes in acute ischemic stroke: a prospective cohort study. Front Nutr. (2025) 12:1707231. doi: 10.3389/fnut.2025.1707231 41567332 PMC12815849

[8] Wang C Qing Y Chen W Li G A. novel nutritional index as a predictor of mortality in dilated cardiomyopathy: a retrospective study. PeerJ. (2022) 10:e12704. doi: 10.7717/peerj.12704 35111392 PMC8783563

[9] Shi Y Wang X Yu C Zhou W Wang T Zhu L . Association of a novel nutritional index with stroke in Chinese population with hypertension: Insight from the China H-type hypertension registry study. Front Nutr. (2023) 10:997180. doi: 10.3389/fnut.2023.997180 37113292 PMC10126229

[10] He D Wang Z Li J Yu K He Y He X . Changes in frailty and incident cardiovascular disease in three prospective cohorts. Eur Heart J. (2024) 45:1058–68. doi: 10.1093/eurheartj/ehad885 38241094

[11] Zhang R Hong J Wu Y Lin L Chen S Xiao Y. Joint association of triglyceride glucose index (TyG) and a body shape index (ABSI) with stroke incidence: a nationwide prospective cohort study. Cardiovasc Diabetol. (2025) 24:7. doi: 10.1186/s12933-024-02569-5 39762919 PMC11705842

[12] Rogers NT Marshall A Roberts CH Demakakos P Steptoe A Scholes S. Physical activity and trajectories of frailty among older adults: evidence from the english longitudinal study of ageing. PloS ONE. (2017) 12:e0170878. doi: 10.1371/journal.pone.0170878 28152084 PMC5289530

[13] Park Y Kim NH Kwon TY Kim SG. A novel adiposity index as an integrated predictor of cardiometabolic disease morbidity and mortality. Sci Rep. (2018) 8:16753. doi: 10.1038/s41598-018-35073-4 30425288 PMC6233180

[14] Fan L Su Y Chen Y Xu L Huang H Lu C . Triglyceride-cholesterol-body weight index associated with the risk of metabolic dysfunction-associated steatotic liver disease: a population-based cross-sectional study. Front Nutr. (2025) 12:1698297. doi: 10.3389/fnut.2025.1698297 41245409 PMC12611659

[15] Bays HE Kirkpatrick CF Maki KC Toth PP Morgan RT Tondt J . Obesity, dyslipidemia, and cardiovascular disease: a joint expert review from the obesity medicine association and the national lipid association 2024. J Clin Lipidol. (2024) 18:e320–50. doi: 10.1016/j.jacl.2024.04.001 38664184

[16] Wazir M Olanrewaju OA Yahya M Kumari J Kumar N Singh J . Lipid disorders and cardiovascular risk: a comprehensive analysis of current perspectives. Cureus. (2023) 15:e51395. doi: 10.7759/cureus.51395 38292957 PMC10825376

[17] Mitchell CC Wilbrand SM Hess T Riesenberg A Danforth D Wesley UV . Cerebrovascular risk factors for body mass index, diabetes, and atherosclerosis in a wisconsin native american population: a cross-sectional observation study. J.Am.Heart Assoc. (2026) 15:e043224. doi: 10.1161/JAHA.125.043224 41778537 PMC13055689

[18] Bluher M. Obesity: global epidemiology and pathogenesis. Nat Rev Endocrinol. (2019) 15:288–98. doi: 10.1038/s41574-019-0176-8 30814686

[19] Hasan B Nayfeh T Alzuabi M Wang Z Kuchkuntla AR Prokop LJ . Weight loss and serum lipids in overweight and obese adults: a systematic review and meta-analysis. J Clin Endocrinol Metab. (2020) 105:dgaa673 doi: 10.1210/clinem/dgaa673 32954416

[20] Lau LH Lew J Borschmann K Thijs V Ekinci EI. Prevalence of diabetes and its effects on stroke outcomes: a meta-analysis and literature review. J Diabetes Investig. (2019) 10:780–92. doi: 10.1111/jdi.12932 PMC6497593 30220102

[21] Saltiel AR Olefsky JM. Inflammatory mechanisms linking obesity and metabolic disease. J Clin Invest. (2017) 127:1–4. doi: 10.1172/JCI92035 28045402 PMC5199709

[22] Deutz NE Bauer JM Barazzoni R Biolo G Boirie Y Bosy-Westphal A . Protein intake and exercise for optimal muscle function with aging: recommendations from the ESPEN Expert Group. Clin Nutr. (2014) 33:929–36. doi: 10.1016/j.clnu.2014.04.007 24814383 PMC4208946

[23] Hill JO Wyatt HR Peters JC. Energy balance and obesity. Circulation. (2012) 126:126–32. doi: 10.1161/CIRCULATIONAHA.111.087213 22753534 PMC3401553

[24] He G Zhang Z Wang C Wang W Bai X He L . 5 million adults in China. Lancet Reg Health West Pac. (2024) 49:101135. doi: 10.1016/j.lanwpc.2024.101135 39050982 PMC11263946

[25] Zhang Z Zhao L Lu Y Meng X Zhou X. Relationship of triglyceride-glucose index with cardiometabolic multi-morbidity in China: evidence from a national survey. Diabetol Metab Syndr. (2023) 15:226. doi: 10.1186/s13098-023-01205-8 37926824 PMC10626797

[26] Pepe MS Janes H Longton G Leisenring W Newcomb P. Limitations of the odds ratio in gauging the performance of a diagnostic, prognostic, or screening marker. Am J Epidemiol. (2004) 159:882–90. doi: 10.1093/aje/kwh101 15105181

[27] Royston P Altman DG Sauerbrei W. Dichotomizing continuous predictors in multiple regression: a bad idea. Stat Med. (2006) 25:127–41. doi: 10.1002/sim.2331 16217841

[28] Carazo-Diaz C Prieto-Valiente L. The dramatic loss of statistical power when dichotomising continuous variables. Rev Neurol. (2024) 78:27–9. doi: 10.33588/rn.7801.2023163 38112654 PMC11064942

[29] Morera OF Dane'el MI Smith BA Redelfs AH Ruiz SL Preacher KJ . Discretizing continuous variables in nutrition and obesity research: a practice that needs to be cut short. Nutr Diabetes. (2023) 13:20. doi: 10.1038/s41387-023-00248-0 37938224 PMC10632499

[30] Greenland S. Dose-response and trend analysis in epidemiology: alternatives to categorical analysis. Epidemiology. (1995) 6:356–65. doi: 10.1097/00001648-199507000-00005 7548341

[31] Desquilbet L Mariotti F. Dose-response analyses using restricted cubic spline functions in public health research. Stat Med. (2010) 29:1037–57. doi: 10.1002/sim.3841 20087875

[32] Zhang Z Zhao L Lu Y Meng X Zhou X. Association between Chinese visceral adiposity index, risk of stroke incidence in middle-aged, et al. J Transl Med. (2023) 21:518. doi: 10.1186/s12967-023-04309-x 37525182 PMC10391837

