# Supplementary material for: Baseline triglyceride–cholesterol–body weight index and risk of incident cardiovascular disease: evidence from the CHARLS and ELSA cohorts
Source: Front Nutr. 2026 Apr 21;13:1807288. doi: 10.3389/fnut.2026.1807288 (PMC13138991; doi:10.3389/fnut.2026.1807288)
Supplement: Supplementary file 1 [file Table_1.docx]

| **Table S1** Baseline characteristics of included and excluded participants in the CHARLS cohort | | | |
| --- | --- | --- | --- |
| **Characteristic** | **Excluded** N = 22,098*^1^* | **Included** N = 3,471*^1^* | **p-value***^2^* |
| **Age(year)** | 58.59 ± 10.32 | 58.12 ± 9.54 | 0.12 |
| **Sex, n(%)** |  |  | <0.001 |
| Male | 10,769 (49%) | 1,556 (45%) |  |
| Female | 11,321 (51%) | 1,915 (55%) |  |
| **Education levels** |  |  | <0.001 |
| Less than lower secondary | 18,858 (86%) | 3,156 (91%) |  |
| Upper secondary & vocational training | 2,505 (11%) | 287 (8.3%) |  |
| Tertiary | 691 (3.1%) | 28 (0.8%) |  |
| **Marital status** |  |  | <0.001 |
| Married or partnered | 12363(86.9%) | 3069(88.4%) |  |
| Other | 1845(13.1%) | 402(11.6%) |  |
| **Current smoking** |  |  | 0.5 |
| No | 9,571 (71%) | 2,454 (71%) |  |
| Yes | 3,848 (29%) | 1,017 (29%) |  |
| **Drinking** |  |  | >0.9 |
| Never | 8,591 (61%) | 2,119 (61%) |  |
| Ever | 5,462 (39%) | 1,352 (39%) |  |
| **Physical activity** |  |  | <0.001 |
| Sedentary | 463 (14%) | 350 (10%) |  |
| Mild | 958 (28%) | 730 (21%) |  |
| Moderate | 1,014 (30%) | 1,038 (30%) |  |
| Vigorous | 993 (29%) | 1,353 (39%) |  |
| **WWI** | 11.11 (10.55, 11.70) | 11.11 (10.56, 11.71) | 0.8 |
| **HDL Cholesterol (mg/dl)** | 50.56 ± 15.40 | 51.48 ± 15.15 | 0.002 |
| **LDL Cholesterol (mg/dl)** | 115.86 ± 35.38 | 116.30 ± 33.82 | 0.6 |
| **Hypertension** |  |  | <0.001 |
| No | 5,872 (52%) | 2,154 (62%) |  |
| Yes | 5,442 (48%) | 1,317 (38%) |  |
| **Diabetes** |  |  | <0.001 |
| No | 6,361 (77%) | 2,914 (84%) |  |
| Yes | 1,935 (23%) | 557 (16%) |  |
| *^1^*Mean ± SD; n (%); Median (Q1, Q3) | | | |
| *^2^*Wilcoxon rank sum test; Pearson's Chi-squared test  WWI, weight-adjusted waist index | | | |
