# Supplementary material for: Baseline triglyceride–cholesterol–body weight index and risk of incident cardiovascular disease: evidence from the CHARLS and ELSA cohorts
Source: Front Nutr. 2026 Apr 21;13:1807288. doi: 10.3389/fnut.2026.1807288 (PMC13138991; doi:10.3389/fnut.2026.1807288)
Supplement: Supplementary file 2 [file Table_2.docx]

| **Table S2** Baseline characteristics of included and excluded participants in the ELSA cohort | | | |
| --- | --- | --- | --- |
| **Characteristic** | **Excluded** N = 17,530*^1^* | **Included** N = 2,272*^1^* | **p-value***^2^* |
| **Age(year)** | 66.49 ± 11.21 | 63.17 ± 7.13 | <0.001 |
| **Sex, n(%)** |  |  | 0.3 |
| Male | 7,957 (45%) | 1,055 (46%) |  |
| Female | 9,573 (55%) | 1,217 (54%) |  |
| **Education levels** |  |  | <0.001 |
| Less than lower secondary | 5,805 (37%) | 748 (33%) |  |
| Upper secondary & vocational training | 7,302 (46%) | 1,161 (51%) |  |
| Tertiary | 2,600 (17%) | 363 (16%) |  |
| **Marital status** |  |  | <0.001 |
| Married or partnered | 4824(67.5%) | 1761(77.5%) |  |
| Other | 2331(32.5%) | 511(22.5%) |  |
| **Current smoking** |  |  | <0.001 |
| No | 5,987 (84%) | 1,968 (87%) |  |
| Yes | 1,167 (16%) | 304 (13%) |  |
| **Drinking** |  |  | <0.001 |
| Never | 726 (12%) | 157 (6.9%) |  |
| Ever | 5,146 (88%) | 2,115 (93%) |  |
| **Physical activity** |  |  | <0.001 |
| Sedentary | 696 (9.9%) | 72 (3.2%) |  |
| Mild | 1,250 (18%) | 223 (9.8%) |  |
| Moderate | 3,345 (48%) | 1,147 (50%) |  |
| Vigorous | 1,736 (25%) | 830 (37%) |  |
| **WWI** | 11.05 (10.53, 11.56) | 10.84 (10.36, 11.28) | <0.001 |
| **HDL Cholesterol (mg/dl)** | -2.19 ± 5.52 | 1.57 ± 0.38 | <0.001 |
| **LDL Cholesterol (mg/dl)** | -1.13 ± 6.42 | 3.83 ± 0.95 | <0.001 |
| **Hypertension** |  |  | <0.001 |
| No | 13,464 (77%) | 1,104 (49%) |  |
| Yes | 4,066 (23%) | 1,168 (51%) |  |
| **Diabetes** |  |  | 0.047 |
| No | 16,721 (95%) | 2,188 (96%) |  |
| Yes | 809 (4.6%) | 84 (3.7%) |  |
| *^1^*Mean ± SD; n (%); Median (Q1, Q3) | | | |
| *^2^*Wilcoxon rank sum test; Pearson's Chi-squared test  WWI, weight-adjusted waist index | | | |
